# Smoothing centre-of-mass tracking to quantify body oscillations and ambulation in broilers

**DOI:** 10.1017/awf.2026.10092

**Published:** 2026-07-31

**Authors:** Rosie H. Whittle, Shawna L. Weimer

**Affiliations:** Department of Poultry Science, University of Arkansas, Fayetteville, AR, USA

**Keywords:** Animal welfare, automated gait analysis, broiler, centre-of-mass tracking, chicken locomotion

## Abstract

Lameness is a key welfare issue in broilers, and the development of non-invasive, automated methods to identify impaired mobility is important for broiler welfare assessment. Current methods for assessing lameness in broilers can be invasive and often require broilers to walk, which may induce stress or pain in lame birds. The aim of this study was to develop a computational approach to quantify broiler ambulation by tracking broilers in an open field and exploring correlations between smoothed movement metrics and bodyweight. At 28 days of age, male broilers (n = 46) were video-recorded during a 180 s open field test. The centre-of-mass was tracked using EthoVision® XT17 at 15 Hz. Zigzag trajectories were detected in 22 broilers and smoothed in RStudio using a 40-point moving average (~2.7 s window). The Euclidean distance (EuD) was calculated between the raw zigzag and smoothed trajectories, and steps were classified based on the variation in EuD over time using a > 2 cm noise threshold. Computed steps were compared with observer counts from the video. Smoothing zigzag trajectories reduced the estimated distance by 57.04% and speed by 56.14%. Computed step counts were 23.49% lower than observer counted steps. There was a strong positive association between raw and smoothed distance, speed, and observer-counted vs computed steps. Bodyweight did not correlate significantly with movement metrics. Smoothing may reduce the influence of lateral body oscillations on estimated distance and speed, although the computational step-detection method requires further refinement to reduce underprediction.

## Introduction

Walking ability in commercial broiler chickens is a critical indicator of welfare and overall health. Impaired mobility can lead to altered behaviour (Weeks *et al.* 2000), stress (Weimer *et al.* 2021), pain (McGeown *et al.* 1999; Danbury *et al.* 2000), reduced feed and water intake (Butterworth *et al.* 2002), and increased susceptibility to disease (McNamee *et al.* 1998). Current assessment methods of broiler walking ability are intrusive, often requiring experimenters to gather and coax the birds to walk, which in turn may alter their natural behaviour and mobility results (Fodor *et al.* 2025; van der Sluis *et al.* 2026). Moreover, gait scoring, the gold standard, can also be subjective and require intensive training and retraining to maintain intra- and inter-observer reliability (Wurtz & Riber 2024).

Technological approaches have emerged to improve the objectivity of broiler mobility assessment. For example, an ultra-wide band tracking system showed that broilers with poorer gait tended to travel shorter distances (van der Sluis *et al.* 2021). Accelerometers can capture overall activity patterns of broilers; however, they may lack the sensitivity to distinguish differences in gait scores (Pearce *et al.* 2023). Further, wearable sensors can be costly, require maintenance, and may affect bird comfort. Non-intrusive measures have also been explored, such as video-based optical flow analysis of broiler flocks, has shown that lower mean variance in flock movement has been associated with a higher prevalence of poor gait (Dawkins *et al.* 2009).

Automated video-based tracking offers a non-invasive alternative for monitoring locomotion patterns in broilers. Previous studies have used such approaches to assess pose estimation (Fodor *et al.* 2023), inactivity metrics, such as latency-to-lie and the number of lying events (Aydin 2017a) and activity metrics, such as step frequency, speed, and step length, as well as body oscillation (Aydin 2017b; Li *et al.* 2023; Fodor *et al.* 2025; van der Sluis *et al.* 2026). However, these approaches focused primarily on inactivity or general oscillatory characteristics and may not capture oscillation magnitude or clearly distinguish oscillatory motion from forward locomotion.

To address this gap, the aim of the present study was to develop a computational approach to quantify the steps of broilers during an open field test. To accomplish this, zigzag trajectories obtained from automated tracking were compared with smoothed pathways derived from centre-of-mass (CoM) tracking, and steps were quantified by using the Euclidean distance between these trajectories. In addition, correlations between smoothed locomotion metrics and bodyweight were examined. We hypothesised that heavier broilers would take fewer steps and exhibit greater CoM deviations compared with lighter birds.

## Materials and methods

All procedures in this study received ethical approval from the Institutional Animal Care and Use Committee at the University of Arkansas Division of Agriculture prior to commencement of the study. Male Cobb 500 broilers were housed in 24 pens (1.2 × 0.91 m; length × width) containing wood-shavings litter, with 12 broilers per pen. On day 28 of age, 46 birds were removed from their home pens and placed individually in a fully enclosed arena (3.6 × 2.4 m) containing fresh wood-shavings litter for an open field test. Each test lasted 180 s, after which bodyweight (g) was recorded before the bird was returned to the home pen. Video recordings were imported into EthoVision® XT17 (Noldus, Wageningen, the Netherlands) for analysis. Broilers were detected in the video using dynamic subtraction, whereby pixels brighter than the background were identified as the animal. Position within the arena was quantified by detecting and tracking the centre of the identified pixels, defined as the centre-of-mass (CoM). Activity threshold and background noise filter settings were adjusted to reduce camera artifacts.

**Figure 1.**
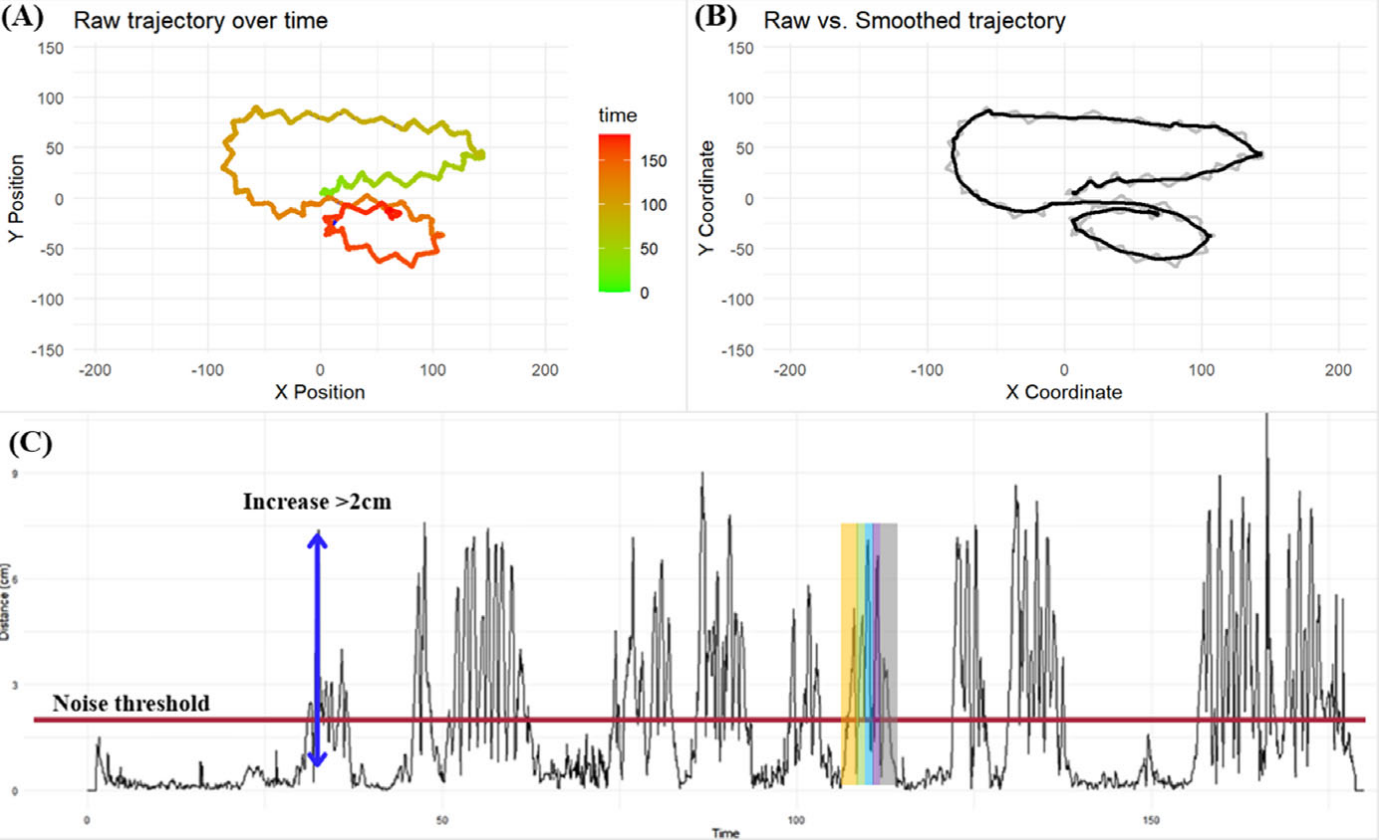
Broiler ambulation coordinate plots from an open field test for the (A) raw trajectory over time, (B) 40-point moving average smoothed trajectory (black line) overlaying the raw trajectory (grey zigzag), and (C) the plotted Euclidean distance between sequential coordinates over time; a ‘step’ was defined as an increase in Euclidean distance greater than 2 cm (blue arrow) from the initial deviation point to the subsequent peak. The highlighted section indicates a series of five steps, where each colour indicates the start and end of each step. Figure 1 long description.

Distance travelled and speed from the raw tracking data were calculated in EthoVision® and extracted for each bird. Raw coordinate data were also exported from EthoVision® and plotted in RStudio. The oscillating nature of the broiler gait produced zigzag patterns in the CoM trajectories (Figure 1A). To ensure sufficient data for analysis, birds that took at least five consecutive steps were included. Zigzag trajectories were identified in 22 broilers, and these data were further analysed.

Coordinate data sampled at 15 Hz were smoothed using a 40-point moving average algorithm (~2.7 s window; Figure 1B). The Euclidean Distance (EuD) between the raw zigzag and smoothed coordinates was calculated as:

Euclidean Distance=sqrtx2−x1^2+y2−y1^2

The distance travelled and speed were then calculated from the smoothed coordinates. Raw, smoothed, and EuD data were extracted into Microsoft Excel® for each bird. An EuD threshold of 2 cm was applied to classify steps, to reduce noise and exclude movements that were not considered steps. The EuD data were then visually inspected, and the start and end times of each peak were defined as the EuD minimum immediately before and after the peak, respectively (Figure 1C). For each step, CoM deviation was defined as the difference between the maximum EuD and the EuD at the onset of the deviation peak, with deviations required to exceed 2 cm (Figure 1C).

Oscillation velocity was defined as the rate of step-related CoM oscillation rather than forward locomotor speed. This metric was included to quantify body oscillation during stepping and may provide information regarding locomotor stability. Greater CoM deviation and higher oscillation velocity could indicate altered balance during walking. Oscillation velocity was calculated as:

$$\text{Oscillation velocity}=\frac{\text{Centre of Mass deviation}}{\left(\text{Step}\;\mathrm{End}\;\text{Time}-\text{Step Start Time}\right)}$$

The number of steps by each broiler was counted from the video by a trained observer and compared with the computational step count.

### Statistical analysis

Data were analysed in R v4.4.1 and Rstudio (2024.04.2+764). Paired *t*-tests were conducted to compare raw and smoothed distance and speed, and observer-counted and computationally derived step counts. A two-sample *t*-test compared the bodyweight of included and excluded broilers. Pairwise correlations between raw and smoothed variables were calculated using Pearson’s correlation coefficient and *P*-values were generated using the ‘Hmisc’ package (Harrell Jr 2003). The resulting correlation matrix was visualised with hierarchical clustering (Ward’s method) to group related variables using ‘ggcorrplot’ package (Kassambara 2016), and the same ordering was applied to the associated *P*-value matrix. *P*-values were displayed in the matrix, with a threshold of *P* < 0.05 used to indicate statistical significance, a tendency was considered when 0.05 < *P* < 0.10.

## Results

**Figure 2.**
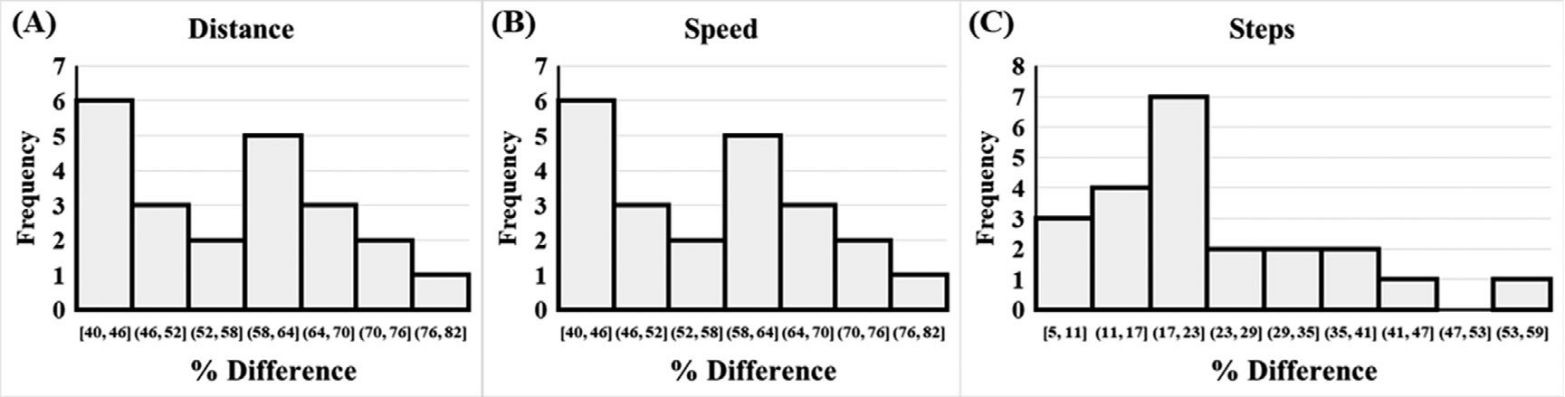
Frequency distribution plots for the percentage difference between (A) raw zigzag trajectories resulting from oscillatory broiler gait and the calculated distance from the smoothed trajectory, (B) raw and smoothed speed, and (C) the percentage difference between the number of steps observed from video and the number of steps calculated from the Euclidean distance between trajectories, collected during an open field test (n = 22). Figure 2 long description.

Of the 46 broilers tested in the open field arena, 47.8% (22 birds) walked sufficiently to produce zigzag trajectories suitable for analysis. Broilers used in this analysis weighed 1,646 (± 29.23) g and ranged from 1,375 to 1,981 g and did not differ from excluded broilers, which weighed 1,628 (± 30.15) g and ranged from 1,268 to 1,860 g (*t* = 0.44, df = 45; *P* = 0.66). Table 1 summarises the differences between raw and smoothed distance and speed, as well as the differences between observer-counted and computated steps. Smoothing the zigzag trajectories reduced estimated distance travelled in the open field arena by 57.04% (*t* = 9.80, df = 21; *P* < 0.001) and reduced estimated speed by 56.14% (*t* = 9.76, df = 21; *P* < 0.001). The percentage difference between raw and smoothed trajectories ranged from 40.41–76.77% for distance (Figure 2A) and 40.42–76.85% for speed (Figure 2B).

**Table 1.** Comparing raw distance and speed derived from the oscillating zigzag gait trajectories of broilers in an open field test (n = 22) with corresponding values calculated from smoothed trajectories, and comparison of steps counted through video observation compared with steps calculated from Euclidean distance trajectory analysis Table 1. long description.

	Raw mean (± SEM)	Smoothed mean (± SEM)	Difference^1^ (%)	Min Difference (%)	Max Difference (%)
Distance (cm)	1,181.97 (± 132.52)	534.94 (± 75.90)	57.04 (± 2.27)	40.41	76.77
Speed (cm s^–1^)	6.58 (± 0.74)	2.97 (± 0.42)	56.14 (± 2.33)	40.42	76.85
Steps (n)	52.05 (± 6.95)	39.14 (± 4.93)	23.49 (± 2.53)	4.76	56.00

1 Difference = Raw – smoothed means.

Computed step counts were 23.49% lower than observer-counted steps (*t* = 4.84, df = 21; *P* = 0.001), with differences ranging from 4.76-56.00% (Figure 2C). Mean CoM deviation per step was 3.97 (± 0.12) cm and ranged from 2.93 to 5.28 cm. While a 2-cm threshold was used to classify steps, the minimum observed CoM deviation exceeded this threshold, indicating that the observed range was not constrained by the threshold criterion. Mean oscillation velocity was 1.27 (± 0.07) cm s^–1^ and ranged from 0.41 to 1.89 cm s^–1^.

**Figure 3.**
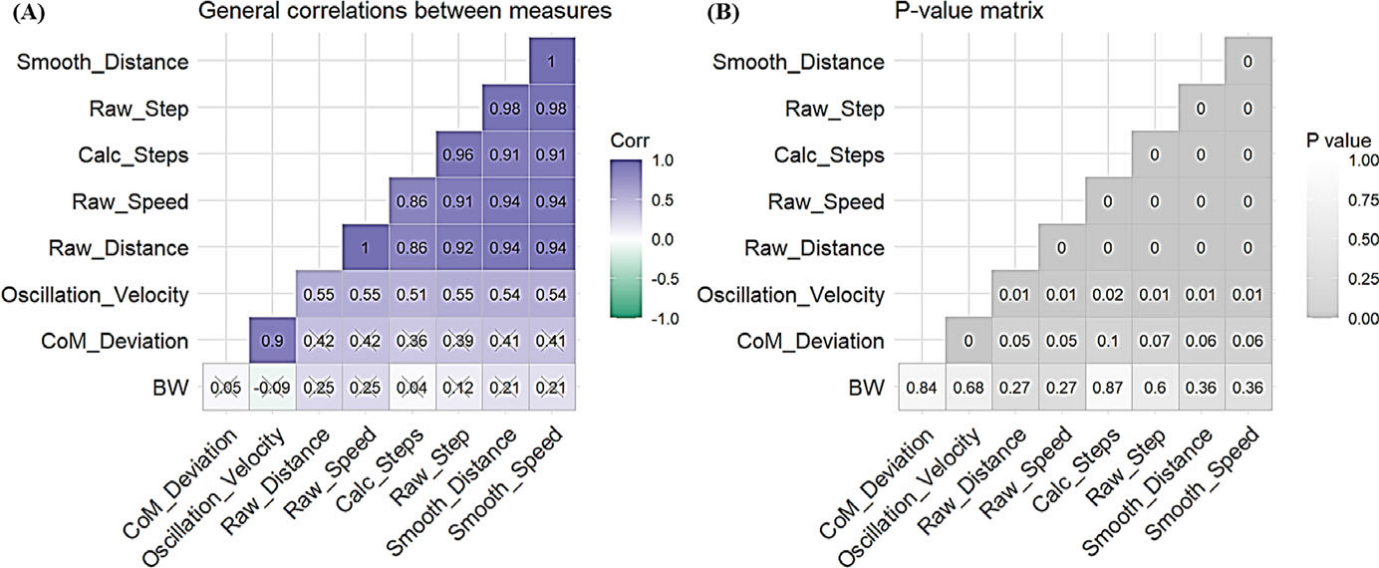
(A) Correlation matrix between the raw and calculated values derived from smoothing zigzag trajectories resulting from oscillatory broiler gait during an open field test (n = 22) and broiler bodyweight. Purple shading indicates positive correlations, while green shading indicates negative correlations. An ‘X’ within a cell indicates non-significant correlations. (B) Corresponding *P*-value matrix for the correlations shown in panel A; a 0 value indicates a *P* < 0.001. Figure 3 long description.

Correlations among raw and smoothed variables are shown in Figure 3. There was a strong positive association between raw and smoothed distance (*r* = 0.94; *P* < 0.001), speed (*r* = 0.94; *P* < 0.001), and steps (*r* = 0.96; *P* < 0.001). Distance, speed, and step-based metrics formed a tight cluster (Figure 3A) of strongly intercorrelated values (*P* < 0.001), with *r* values ranging between 0.86 and 0.98. Speed and distance metrics showed moderate positive correlations with average CoM deviation (*r* = 0.36–0.42; *P* = 0.05–0.10). Bodyweight did not significantly correlate with any raw or smoothed movement measures (*r* < 0.30; *P* > 0.05).

## Discussion

Smoothing the broiler trajectories reduced distance and speed by 57%, while strong correlations between the raw and smoothed values indicated that the overall movement pattern was preserved. This suggests that smoothing reduced the contribution of body oscillations to distance and speed estimates and may therefore provide a measure that better characterises net forward locomotion. This distinction is important because the automated tracking software used in this study was developed for small laboratory animals and does not account for the oscillatory gait characteristics of broilers. The results of the current study suggest that smoothing may improve the interpretability of movement metrics derived from centre-of-mass tracking in broilers.

Slightly less than half of the broilers tested exhibited sufficient voluntary movement to generate zigzag trajectories for analysis, highlighting a key limitation of open field tests, as broilers with poorer gaits or reduced motivation to walk may be underrepresented (Wurtz & Riber 2024). Open field behaviour may also be influenced by social isolation stress, which can reduce movement and bias results toward less fearful individuals (Suarez & Gallup 1983). Interestingly, there was no difference in bodyweight between broilers included or excluded as they did not walk sufficiently in this study, indicating that willingness to walk in the arena may not have been explained by bodyweight alone. Instead, both walking ability and behavioural responses to social isolation may have influenced which birds were represented in the final dataset.

The results should also be considered in the context of previous work showing that heavier broilers differ from lighter birds in gait properties such as speed, force, and propulsion (Corr *et al.* 2007), and that the energetic cost of locomotion increases with bodyweight (Tickle *et al.* 2018). Therefore, it is plausible that the magnitude of body oscillation varies with age, size, or health status, although this remains to be explored. Smoothing may provide a more consistent basis for comparing locomotion across developmental stages or genetic strains by reducing the influence of oscillatory motion on conventional movement metrics.

In addition to distance and speed metrics, using Euclidean distance and centre-of-mass deviations to estimate step counts was strongly correlated with observer-counted steps, although step number was consistently underpredicted. This pattern suggests that the conservative 2 cm noise threshold used to identify steps may have reduced false positives (Type I errors) but increased false negatives (Type II errors), resulting in missed true steps. The underprediction observed indicates that further refinement of the step-detection algorithm is needed before this approach can be used as an automated estimate of the number of steps.

Movement-magnitude metrics (distance, speed, and steps) were highly intercorrelated, indicating that they captured overlapping aspects of locomotor activity. In contrast, centre-of-mass deviation was only moderately correlated with distance and speed, suggesting that it may reflect lateral adjustments associated with postural control or balance rather than forward progression alone, which may be particularly relevant in broilers because body conformation can affect gait mechanics (Caplen *et al.* 2012). Similarly, oscillation velocity may provide complementary information on the contribution of body oscillation to step rate. However, because centre-of-mass deviation and oscillation velocity are mathematically related, these measures should not be interpreted as statistically independent.

Bodyweight was not significantly associated with movement in this dataset. This does not necessarily contradict previous studies demonstrating positive relationships between bodyweight, gait score, and body oscillations (Caplen *et al.* 2012; Fodor *et al.* 2025; van der Sluis *et al.* 2026), but instead suggests that these relationships were not yet strongly expressed in broilers used in this study. It is also important to note that the ability to detect oscillatory patterns depends on the bird walking long enough to generate trajectories that are suitable for analysis. This may be particularly challenging in birds with poorer gaits, which are often less willing to walk voluntarily (Li *et al.* 2023).

These results highlight a practical trade-off in broiler locomotion studies. Younger and lighter broilers may be more likely to walk voluntarily, making data collection easier, whereas older and heavier broilers may show gait impairment differences more clearly but may be less willing to walk in voluntary tests. Forced walking tests on runways or treadmills may overcome this limitation, but introduce other sources of bias in walking metrics, such as the number of steps taken (van der Sluis *et al.* 2026). An alternative approach is to monitor voluntary walking *in situ*, such as when broilers walk between feeders and drinkers, though tracking individuals in large groups remains technically challenging.

### Future directions

To validate its predictive value for lameness detection, future research should compare this computational approach with bird-level measures of walking ability (i.e. gait scores). Further refinement and automation of the algorithm are also needed to reduce processing time and improve step detection accuracy, particularly for broilers with subtle gait alterations. Other smoothing approaches, such as spline smoothing, should also be explored to determine whether they perform better than moving averages in birds with faster or irregular trajectories. Ultimately, this method could be further developed for real-time monitoring of walking ability in commercial broiler systems.

### Animal welfare implications

There is a clear need for objective, automated alternatives to traditional gait scoring in broilers, which can be subjective and prone to under- or overestimating the prevalence of gait abnormalities within a flock. Current methods for assessing gait and walking ability in broilers often require birds to be disturbed, handled, or encouraged to walk, or relocated to novel environments – procedures that can induce stress and compromise welfare. Technologies capable of monitoring broiler walking ability within the home environment could reducei stress while enabling more systematic, real-time detection of gait abnormalities at the flock level.

## Conclusions

Smoothing the raw zigzag trajectories derived from centre-of-mass tracking reduced the influence of oscillatory motion on estimated distance and speed, while preserving the underlying movement pattern. Although the computed step counts were strongly correlated with observer counts from video, they were consistently underpredicted, indicating the need for further algorithm refinement. Bodyweight did not significantly influence inclusion in the analysis or the locomotion metrics in 28-day-old broilers. Exploring this approach across a broader range of ages, walking abilities, and health conditions will be important for determining its robustness and potential application to automated monitoring of broiler locomotion.

## Figure 1 Long description

Figure 1 illustrates how broiler movement trajectories were processed to quantify steps during an open field test. Panel A shows a raw movement trajectory over time, with color indicating progression through time. Panel B overlays the raw trajectory with a smoothed trajectory generated using a 40-point moving average. Panel C displays Euclidean distance over time, with a horizontal noise threshold marked. Steps are defined as increases in distance greater than 2 cm from an initial deviation point to a subsequent peak. A highlighted section indicates five consecutive steps, with different colors marking the start and end of each step.

Navigate back to Figure 1.

## Table 1. Long description

Table 1 compares locomotor measures for broiler chickens calculated from raw versus smoothed movement trajectories during an open field test. Outcomes include distance traveled (centimeters), speed (centimeters per second), and number of steps. For each outcome the table reports the mean and standard error from raw and smoothed data, the percentage difference between the means and the maximum and minimum differences. Across all measures, smoothed trajectories produced lower values than raw trajectories. Mean percentage differences were 57% for distance traveled, 57% for speed, and 23% for step counts, with wide ranges observed across individuals.

Navigate back to Table 1..

## Figure 2 Long description

Figure 2 shows frequency distributions of percentage differences between movement metrics derived from raw and smoothed broiler trajectories during an open field test (n = 22). Three histograms display results for distance traveled, movement speed, and step counts. Percentage differences for distance and speed are generally larger and span higher ranges than those for step counts, indicating greater effects of trajectory smoothing on distance and speed than on step detection.

Navigate back to Figure 2.

## Figure 3 Long description

Figure 3 presents associations among broiler locomotion measures and bodyweight during an open field test (n = 22). Panel A shows a correlation matrix of raw, smoothed, and calculated gait variables, with color indicating the strength and direction of correlations. Panel B shows the corresponding p‑value matrix, with cells indicating the statistical significance of the correlations. Together, the panels summarize the relationships among distance, speed, step measures, oscillatory gait metrics, and bodyweight.

Navigate back to Figure 3.
